# Serum 25-hydroxyvitamin D concentrations and their impact on all-cause mortality in Parkinson’s disease: insights from National Health and Nutrition Examination Survey 1999–2020 data

**DOI:** 10.3389/fnut.2024.1423651

**Published:** 2024-08-09

**Authors:** Yufei Yong, Hui Dong, Zhen Zhou, Yan Zhu, Meiling Gu, Wenxiao Li

**Affiliations:** Department of Health Management Center, The Affiliated Hospital of Qingdao University, Qingdao, China

**Keywords:** Parkinson’s disease, vitamin D, all-cause mortality, NHANES, nutrition

## Abstract

**Background and purpose:**

This study explores the relationship between serum 25-hydroxyvitamin D [25(OH)D] levels and mortality among Parkinson’s disease (PD) patients, providing evidence for the potential benefits of vitamin D (VD) supplementation.

**Methods:**

PD patients were collected from the National Health and Nutrition Examination Survey (NHANES) database from 1999 to 2020. These patients were categorized based on their serum 25(OH)D levels: deficiency, insufficiency, and sufficiency. We compared demographic information and analyzed mortality data from the National Death Index. A restricted cubic spline model assessed the nonlinear association between 25(OH)D levels and mortality, complemented by multivariable Cox regression analysis. Consistency of results was checked through subgroup analysis.

**Results:**

The study included 364 PD patients: 87 (23.9%) with VD deficiency, 121 (33.2%) with insufficiency, and 156 (42.9%) with sufficiency. Demographically, 46.4% were male, and 56% were over 65 years. The deficiency group predominantly consisted of Mexican Americans (53.1%), had lower income levels, a higher unmarried rate, and increased liver disease incidence. The analysis showed a U-shaped curve between 25(OH)D levels and mortality risk, with the lowest risk at 78.68 nmol/L (*p*-non-linear = 0.007, *p*-overall = 0.008). Kaplan–Meier analysis found the highest survival rates in patients with 25(OH)D levels between 75–100 nmol/L (*p* = 0.039). Compared to this group, patients with levels below 50 nmol/L had a 3.52-fold increased mortality risk (95% CI = 1.58–7.86, *p* = 0.002), and those above 100 nmol/L had a 2.92-fold increase (95% CI = 1.06–8.05, *p* = 0.038). Age-specific subgroup analysis (*p* = 0.009) revealed that both very low (<50 nmol/L) and high (>100 nmol/L) levels increased mortality risk in patients under 65, while levels below 75 nmol/L raised mortality risk in older patients.

**Conclusion:**

Serum 25(OH)D levels are nonlinearly linked to mortality in PD patients, with optimal survival rates occurring at 75–100 nmol/L. Deviations from this range increase the risk of death.

## 1 Introduction

Parkinson’s disease (PD) is the second most common neurodegenerative disorder worldwide, marked by symptoms such as resting tremor, rigidity, bradykinesia, and postural instability. The prevalence of PD has increased significantly, from 0.9 per 1,000 individuals in the 1980s to 3.81 per 1,000 in the period from 2010 to 2023. Among those over 60 years old, the incidence is 9.34 per 1,000. Estimates suggest that by 2030, the global PD population will range between 8.7 and 9.3 million (1, 2). The pathological hallmarks of PD include the accumulation of Lewy bodies and the loss of dopaminergic neurons in the substantia nigra (3). Risk factors for PD encompass older age, male sex, genetic predispositions, exposure to pesticides, concurrent diabetes, and dairy consumption (4, 5), whereas smoking, regular physical activity, and caffeine intake may offer protective effects (6). Research indicates that PD patients experience a 2.22-fold increase in all-cause mortality compared to the general population, with mortality risk increasing by 1.05 times with each additional year of age (7, 8). A report by Public Health England noted a 45% rise in PD-related mortality from 2001 to 2014 (9), highlighting the critical need to address mortality to improve outcomes for PD patients.

Vitamin D (VD), a fat-soluble vitamin, is synthesized in the skin or obtained through dietary supplements. It is converted to 25-hydroxyvitamin D [25(OH)D] in the liver and further hydroxylated in the kidneys to its active form, 1,25-dihydroxyvitamin D (10). Serum levels of 25(OH)D below 50 nmol/L typically indicate VD deficiency (11). A survey utilizing the National Health and Nutrition Examination Survey (NHANES) data in the United States found that 41.6% of adult participants had 25(OH)D levels below this threshold (12). VD deficiency impacts calcium absorption, potentially leading to osteoporosis, and is associated with higher risks of several conditions including cancer, infections, autoimmune and cardiovascular diseases, and various psychological and neurological disorders such as depression, bipolar disorder, schizophrenia, Alzheimer’s disease, and PD. (13–15) The National Academy of Sciences advises a daily intake of 15 micrograms (600 IU) of VD for individuals under 70 years old and 20 micrograms (800 IU) for those aged 70 and above to mitigate these risks (16).

Previous research has shown a significant inverse relationship between serum 25(OH)D levels and PD incidence rates (17). Both VD deficiency [total 25(OH)D <50 nmol/L] and insufficiency [total 25(OH)D <75 nmol/L] increase the risk of PD development (18). However, the association between 25(OH)D levels and mortality rates in PD patients has been less studied. This research utilizes NHANES data to explore the impact of serum 25(OH)D levels on all-cause mortality in PD patients, aiming to provide insights that could improve prognosis for these patients.

## 2 Materials and methods

### 2.1 Study design and participants

The NHANES database, managed by the National Center for Health Statistics (NCHS), assesses the health and nutritional status of the United States population. This study obtained Institutional Review Board approval from the NCHS, with all participants providing written informed consent.

### 2.2 Diagnosis of PD

Participants from the NHANES questionnaire survey who reported using medications such as methyldopa, levodopa, carbidopa, pramipexole, ropinirole, amantadine, selegiline, entacapone, benztropine, trihexyphenidyl, zonisamide, apomorphine, tolcapone, orphenadrine, rasagiline, rotigotine, and safinamide, based on criteria established in previous studies (19–21).

### 2.3 Measurement of serum 25(OH)D

Serum levels of 25(OH)D, including both D2 and D3 variants, were initially measured using the RIA method (NHANES 2001–2006) and subsequently by standardized LC-MS/MS (2007–2018). Data from 2001–2006 were adjusted to align with LC-MS/MS standards for consistency. VD status was classified into three categories: deficiency (<50 nmol/L), insufficiency (50–75 nmol/L), and sufficiency (≥75 nmol/L) (10).

### 2.4 Ascertainment of mortality

Mortality data were sourced from the National Death Index (NDI) and updated through December 31, 2019. The data were linked via the NCHS, with mortality eligibility indicated by the variable ELIGSTAT and vital status by MORTSTAT. Follow-up duration was calculated in person-months from the interview date to either the date of death or the end of the study, tracked by the variable PERMTH_INT. Causes of death were classified according to the 10th revision of the International Statistical Classification of Diseases, Injuries, and Causes of Death (ICD-10).

### 2.5 Assessment of covariates

Demographic information and medical history were collected to serve as covariates. These included age (grouped as ≤65 years and >65 years), sex, body mass index (BMI categorized as <25, 25–30, and ≥30 kg/m^2^), race, education level, marital status, and poverty income ratio (PIR categorized as <1.3, 1.3–3.5, and ≥3.5). Lifestyle factors assessed included caffeine consumption, smoking history (classified into non-smokers (less than 100 cigarettes), former smokers (more than 100 cigarettes but quit), and current smokers (more than 100 cigarettes and currently smoking) based on lifetime cigarette consumption), and alcohol consumption history. Physical activity was determined through a questionnaire, defining moderate activity as at least 10 min of exercise per day over the last 30 days that induced light sweating or a slight to moderate increase in breathing or heart rate.

Health conditions were meticulously recorded. Diabetes was identified by fasting blood glucose levels ≥7 mmol/L, HbA1c ≥11.1 mmol/L, use of diabetes medication, or a previous diagnosis by a doctor. Hypertension was defined by blood pressure ≥140/90 mmHg, use of antihypertensive medication, or a prior diagnosis. The presence of stroke, atherosclerotic cardiovascular disease (ASCVD), liver disease, chronic obstructive pulmonary disease (COPD), cancer, and chronic heart failure were all established based on self-reported medical history.

### 2.6 Statistical analysis

Continuous variables were presented as either medians with interquartile ranges (IQR) or means with standard deviations (SD), and analyzed using the Kruskal–Wallis test. Categorical variables were reported as frequencies and percentages and evaluated using the chi-square test. To handle missing data, multiple imputation techniques were employed.

In the analysis, serum 25(OH)D levels were treated as a continuous variable in Cox regression to assess their linear relationship with mortality. Restricted cubic spline (RCS) curves depicted this relationship, with reference groups selected based on RCS outcomes. Kaplan–Meier survival curves were generated to compare survival rates across different groups using the Breslow test.

Covariates such as age, sex, marital status, race, PIR, education level, and smoking status were included in the multivariable Cox regression analysis (Model 1). Model 2 extended adjustments to include hypertension, diabetes, stroke, ASCVD, and liver disease history, exploring the independent impact of serum 25(OH)D on PD mortality rates.

Subgroup analyses based on age and sex were conducted to ensure result consistency, employing likelihood ratio tests to explore interactions between these subgroups and 25(OH)D levels. All statistical analyses were performed using R software version 4.3.1. A *p*-value of less than 0.05 was considered statistically significant.

## 3 Results

### 3.1 Participant demographics

**Figure 1:**
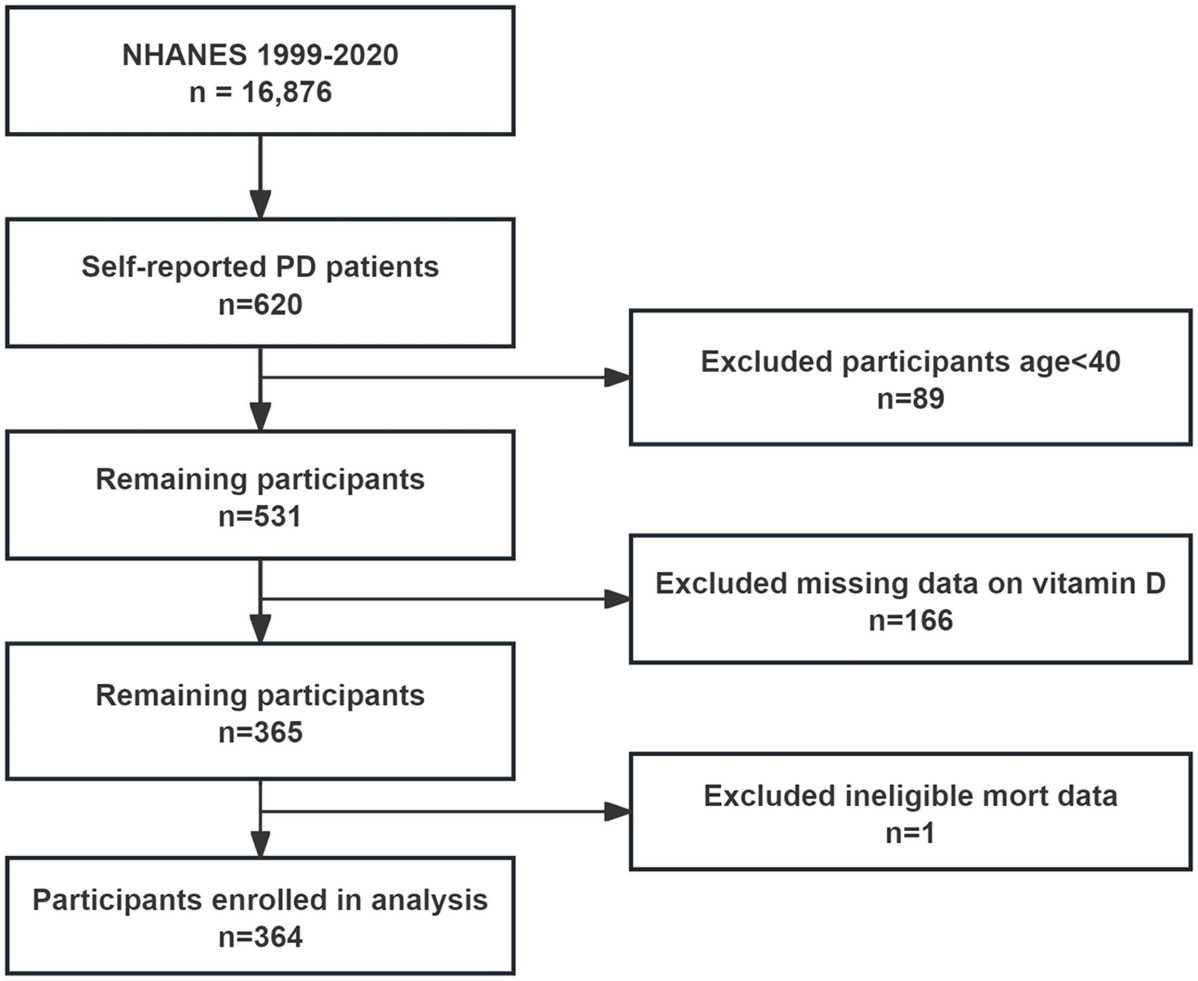
Flow chart of study inclusion.

From the NHANES interviews conducted between 1999 and 2020, 16,876 participants were initially considered, including 620 who self-reported having PD. After excluding 89 individuals under 40 years old and 167 lacking complete serum 25(OH)D and mortality data, 364 participants were included in the final analysis (Figure 1). These participants were categorized based on their serum 25(OH)D levels into deficiency (87 participants, 23.9%), insufficiency (121 participants, 33.2%), and sufficiency (156 participants, 42.9%). Analysis revealed no significant age or gender differences across these groups. Notably, the deficiency group had a higher proportion of Mexican American participants (53.1%), lower poverty income ratios, a greater number of unmarried individuals, and a higher incidence of liver disease (Table 1).

**Table 1:** Basic characteristics of participants.

Characteristics	Deficiency (<50 nmol/L) (*n* = 87)	Insufficiency (50–75 nmol/L) (*n* = 121)	Sufficiency (≥75 noml/L) (*n* = 156)	*p*-value
Age, *n* (%)				0.4
≤65	42 (63%)	54 (59%)	64 (53%)	
>65	45 (37%)	67 (41%)	92 (47%)	
Sex, *n* (%)				0.6
Female	46 (67%)	59 (59%)	90 (64%)	
Male	41 (33%)	62 (41%)	66 (36%)	
Smoking, *n* (%)				0.065
Never	33 (43%)	62 (54%)	82 (55%)	
Former	27 (20%)	39 (27%)	49 (28%)	
Now	27 (37%)	20 (19%)	25 (16%)	
Drinking, *n* (%)				0.2
Never	11 (8.2%)	15 (10%)	20 (9.9%)	
Former	34 (38%)	39 (27%)	45 (24%)	
Now	42 (54%)	67 (63%)	91 (66%)	
Ethnicity, *n* (%)				0.002
Mexican American	17 (8.9%)	7 (2.7%)	8 (1.6%)	
Non-Hispanic White	48 (73%)	81 (81%)	119 (91%)	
Non-Hispanic Black	15 (11%)	15 (7.0%)	17 (4.0%)	
Other race	7 (7.3%)	18 (8.9%)	12 (3.5%)	
Marital status, *n* (%)				0.039
Never married	16 (16%)	15 (12%)	6 (3.0%)	
Widowed/divorced/separated	30 (33%)	48 (41%)	31 (25%)	
Married/living with partner	41 (52%)	58 (48%)	62 (72%)	
PIR, *n* (%)				0.031
<1.3	44 (38%)	43 (27%)	37 (14%)	
1.3–3.5	25 (28%)	48 (43%)	73 (46%)	
≥3.5	18 (34%)	30 (30%)	46 (40%)	
Education level, *n* (%)				0.5
Less than high school	35 (26%)	36 (21%)	39 (16%)	
High school/equivalent	16 (23%)	29 (26%)	30 (22%)	
College/more than high school	36 (51%)	56 (53%)	87 (63%)	
BMI, kg/m^2^, *n* (%)				0.4
<25	23 (20%)	33 (26%)	44 (31%)	
25–28	22 (32%)	35 (26%)	50 (32%)	
≥28	42 (48%)	53 (47%)	62 (37%)	
Caffeine consumption	70 (11, 140)	126 (28, 243)	133 (50, 245)	0.11
Physical activity				0.12
Active	15 (28%)	35 (47%)	46 (34%)	
Inactive	57 (72%)	65 (53%)	93 (66%)	
Diabetes mellitus, *n* (%)				0.5
No	54 (69%)	88 (77%)	108 (76%)	
Yes	33 (31%)	33 (23%)	48 (24%)	
Hyperlipidemia, *n* (%)				0.7
No	21 (24%)	21 (18%)	26 (17%)	
Yes	66 (76%)	100 (82%)	130 (83%)	
Hypertension, *n* (%)				0.3
No	30 (34%)	38 (32%)	51 (42%)	
Yes	57 (66%)	83 (68%)	105 (58%)	
Stroke, *n* (%)				0.4
No	71 (81%)	103 (88%)	139 (90%)	
Yes	16 (19%)	18 (12%)	17 (9.8%)	
ASCVD, *n* (%)				0.06
No	56 (66%)	80 (75%)	120 (84%)	
Yes	31 (34%)	41 (25%)	36 (16%)	
Cancer, *n* (%)				0.6
No	69 (70%)	94 (75%)	124 (78%)	
Yes	18 (30%)	27 (25%)	32 (22%)	
Chronic heart failure, *n* (%)				0.7
No	76 (86%)	107 (91%)	140 (92%)	
Yes	11 (14%)	14 (9.2%)	16 (8.2%)	
COPD, *n* (%)				0.5
No	80 (91%)	104 (85%)	138 (90%)	
Yes	7 (9.2%)	17 (15%)	18 (10%)	
Liver disease, *n* (%)				0.019
No	78 (86%)	117 (98%)	143 (90%)	
Yes	9 (14%)	4 (2.2%)	13 (9.5%)	

PIR, ratio of family income to poverty; BMI, body mass index; ASCVD, arteriosclerotic cardiovascular disease; COPD, chronic obstructive pulmonary disease.

### 3.2 25(OH)D levels and mortality in PD

**Figure 2:**
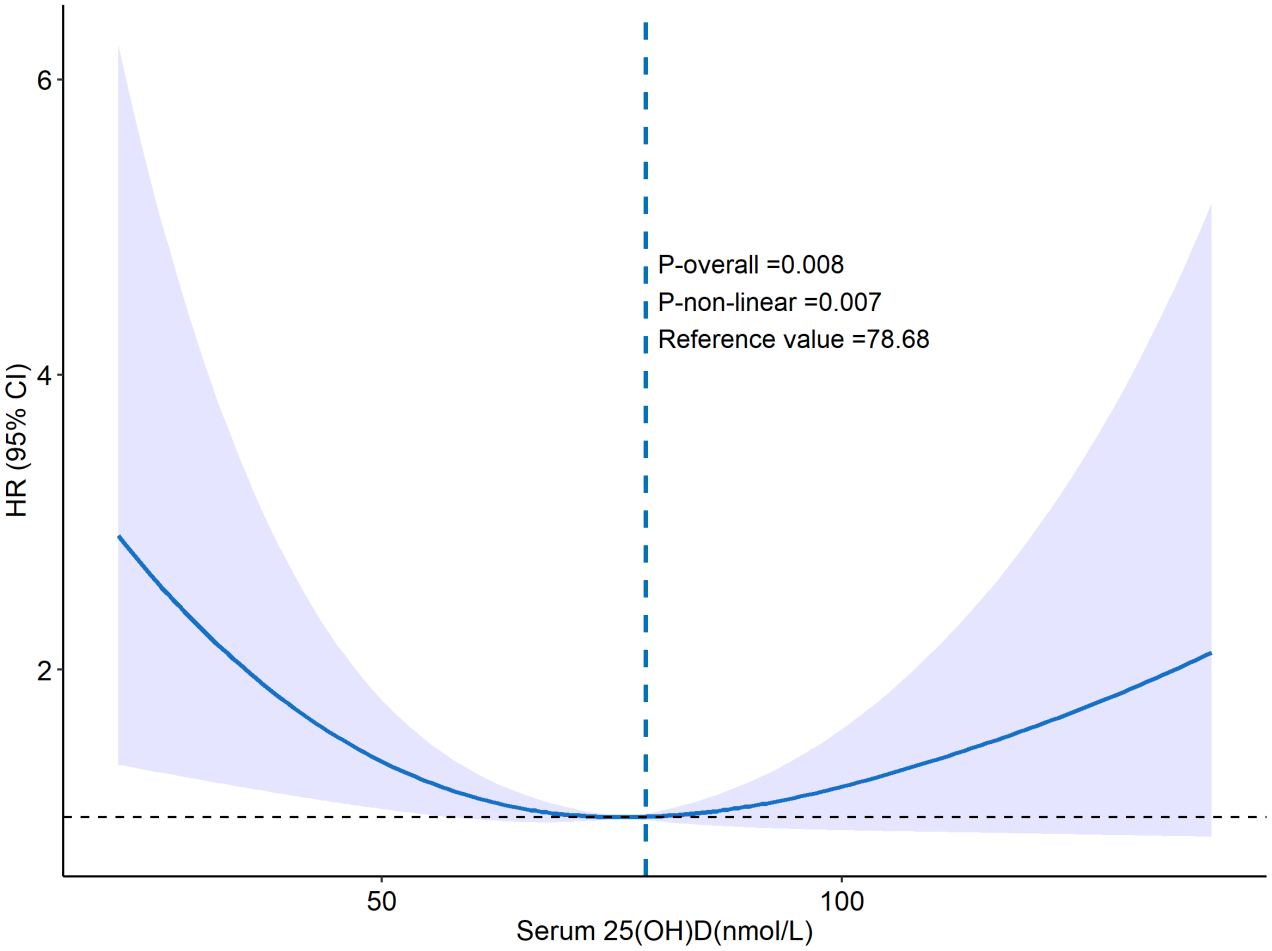
The RCS curve of 25(OH)D and all-cause mortality. Adjusted by age, sex, marital status, ethnicity, smoking, PIR, education level, drinking, BMI, diabetes mellitus, hypertension, hyperlipidemia, COPD, chronic heart failure, stroke, ASCVD, cancer, liver disease.

Over an average follow-up of 8.5 years, 132 PD patients died, primarily from heart disease (24%) and malignant tumors (14%). No significant mortality differences were observed among patients with varying levels of VD (Table 2). Analysis using serum 25(OH)D as a continuous variable in both univariate and multivariate Cox regression revealed no direct correlation with mortality rates among PD patients (Table 3). However, after adjusting for variables such as age, sex, race, marital status, PIR, BMI, smoking habits, and medical conditions including hypertension, diabetes, and stroke, a U-shaped relationship emerged between serum 25(OH)D levels and mortality risk, with the lowest risk observed at 78.68 nmol/L (*p*-non-linear = 0.007, *p*-overall = 0.008) (Figure 2).

**Table 2:** Cause of death in participants.

Characteristic	Overall (*n* = 132)	Deficiency (<50 nmol/L) (*n* = 39)	Insufficiency (50–75 nmol/L) (*n* = 50)	Sufficiency (≥75noml/L) (*n* = 43)	*p*-value
Cause of death					0.5
Diseases of heart	34 (24%)	8 (22%)	13 (25%)	13 (25%)	
Malignant neoplasms	19 (14%)	8 (24%)	6 (14%)	5 (7.3%)	
Chronic lower respiratory diseases	4 (2.2%)	1 (4.4%)	3 (3.0%)	0 (0%)	
Accidents	4 (3.0%)	0 (0%)	1 (1.7%)	3 (6.3%)	
Cerebrovascular diseases	4 (2.7%)	2 (2.5%)	1 (1.0%)	1 (4.5%)	
Alzheimer’s disease	6 (4.0%)	2 (6.9%)	3 (4.9%)	1 (1.4%)	
Diabetes mellitus	3 (3.5%)	1 (3.5%)	1 (3.8%)	1 (3.2%)	
Influenza and pneumonia	3 (2.9%)	1 (4.1%)	2 (5.1%)	0 (0%)	
Nephritis, nephrotic syndrome and nephrosis	1 (0.3%)	0 (0%)	0 (0%)	1 (0.7%)	
All other causes	54 (43%)	16 (33%)	20 (41%)	18 (51%)	

**Table 3:** Relationship between serum 25(OH)D and the all-cause mortality.

Vitamin D	HR	95% CI	*p-*value
Unadjusted	0.99	0.98–1.00	0.200
Model1	0.99	0.98–1.00	0.243
Model2	0.99	0.98–1.01	0.271

Model 1 was adjusted for age, sex, marital status, smoking, drinking, BMI. Model 2 was adjusted for Model 1 + diabetes mellitus, hypertension, COPD, chronic heart failure, stroke, ASCVD, cancer. HR, hazard ratio; CI, confidence intervals; BMI, body mass index; ASCVD, arteriosclerotic cardiovascular disease.

**Figure 3:**
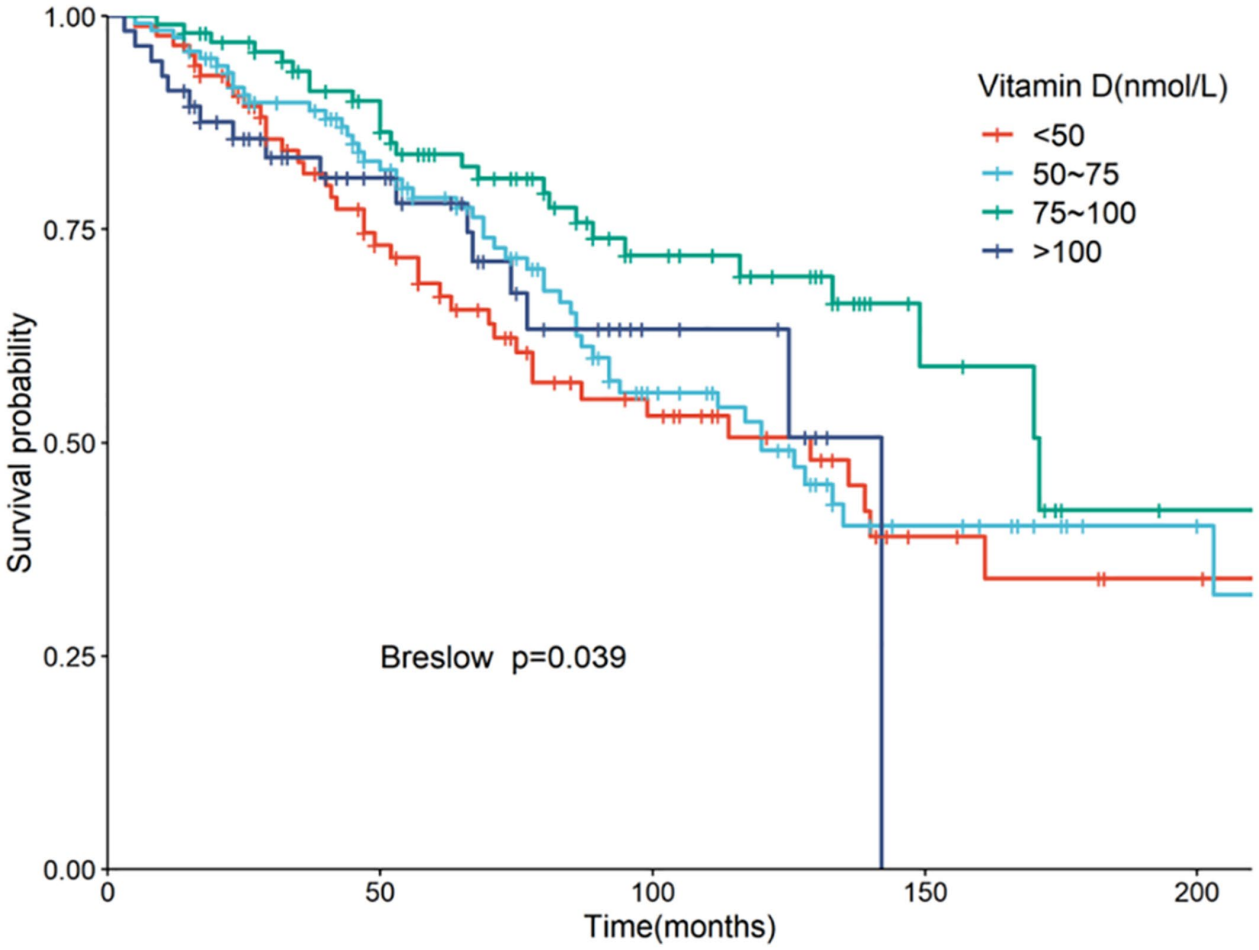
Kaplan–Meier curve of all-cause mortality by four groups of 25(OH)D.

**Figure 4:**
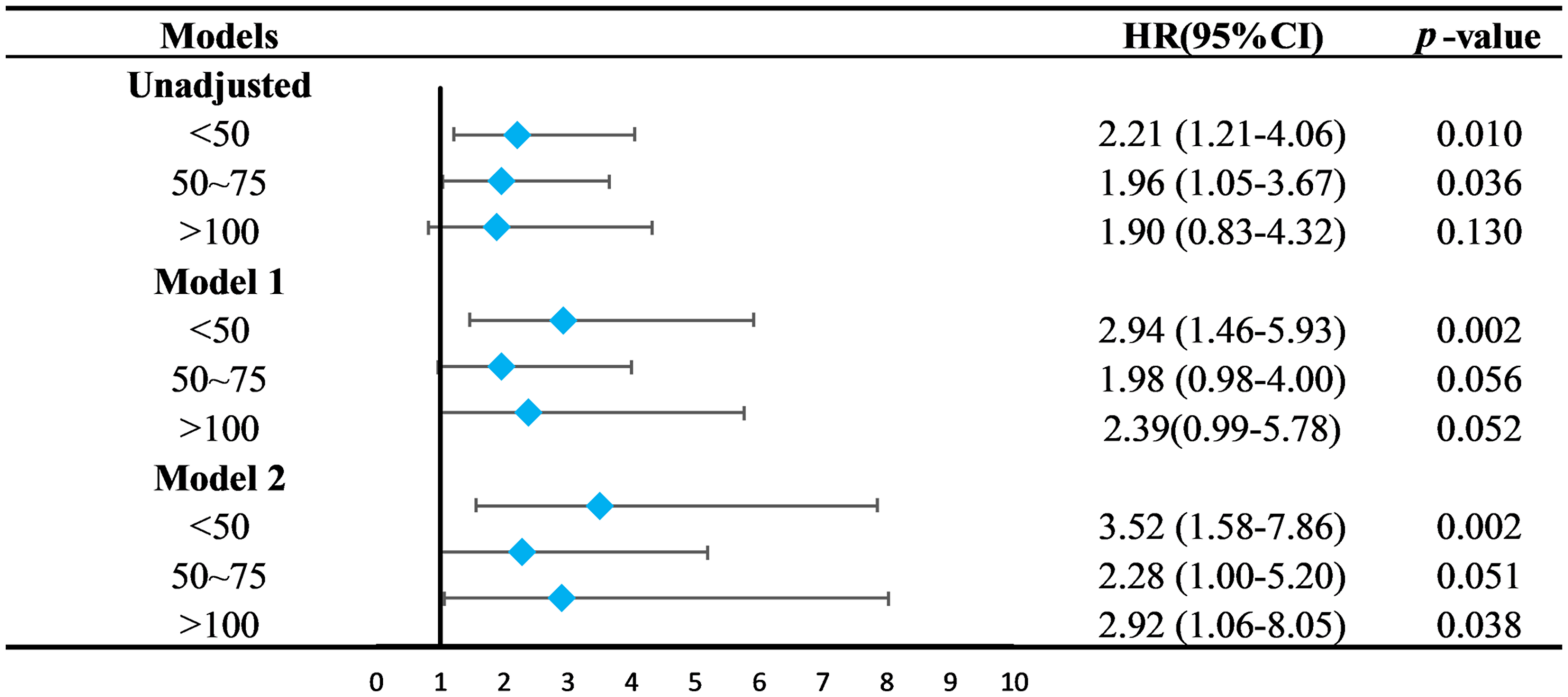
Relationship between 25(OH)D groups and the all-cause mortality. The 75–100 mmol/L group was set as the control group. Model 1 was adjusted for age, sex, marital status, ethnicity, smoking, PIR, education level. Model 2 was adjusted for Model 1 + diabetes mellitus, hypertension, stroke, ASCVD, liver disease. HR, hazard ratio; CI, confidence intervals; PIR, ratio of family income to poverty; ASCVD, arteriosclerotic cardiovascular disease.

Serum 25(OH)D was grouped into four categories: Group 1 (<50 nmol/L), Group 2 (50–75 nmol/L), Group 3 (75–100 nmol/L, the reference group), and Group 4 (>100 nmol/L). Kaplan–Meier analysis showed the highest survival rate in Group 3, with significant differences noted (*p* = 0.039) (Figure 3). Univariate Cox regression analysis results, detailed in Table 4, highlighted the mortality risks associated with different serum 25(OH)D levels. Multivariate COX regression analysis results are shown in Figure 4. Model 1, adjusted for demographic factors like age and sex, indicated that the mortality risk in Group 1 was 2.94 times higher than in Group 3 (95% CI = 1.46–5.93, *p* = 0.002). Model 2 incorporated additional adjustments for covariates including hypertension, diabetes, and history of ASCVD. This model showed that the mortality risk for Group 1 was 3.52 times higher than that of Group 3 (95% CI = 1.58–7.86, *p* = 0.002), and the risk for Group 4 was 2.92 times higher (95% CI = 1.06–8.05, *p* = 0.038).

**Table 4:** Relationship between covariant variables and the all-cause mortality.

Variables	HR (95% CI)	*p*-value
Age (>65)	3.81 (2.33–6.23)	<0.001
Male	2.12 (1.30–3.44)	0.002
Smoking		
Former	1.96 (1.11–3.45)	0.021
Now	1.54 (0.78–3.06)	0.2
Drinking		
Former	0.89 (0.42–1.90)	0.8
Now	0.6 (0.29–1.24)	0.2
Ethnicity		
Non-Hispanic White	0.99 (0.49–2.03)	>0.9
Non-Hispanic Black	0.8 (0.33–1.93)	>0.6
Other race	0.54 (0.14–2.07)	0.4
Marital status		
Widowed/divorced/separated	1.35 (0.68–2.69)	0.4
Married/living with partner	0.6 (0.30–1.20)	0.15
PIR, *n* (%)		
1.3–3.5	0.83 (0.51–1.36)	0.5
≥3.5	0.73 (0.41–1.31)	0.3
Education level		
High school or equivalent	0.41 (0.20–0.85)	0.017
College or more than high school	0.71 (0.43–1.17)	0.2
BMI		
25–28	1.61 (0.89–2.91)	0.12
≥28	0.92 (0.51–1.64)	0.8
Caffeine consumption	1.00 (1.00–1.00)	0.93
Physical activity	0.64 (0.36–1.16)	0.143
Diabetes mellitus, *n* (%)	2.18 (1.32–3.60)	0.002
Hyperlipidemia, *n* (%)	1.22 (0.71–2.07)	0.5
Hypertension, *n* (%)	2.24 (1.35–3.70)	0.002
Stroke, *n* (%)	1.85 (1.02–3.35)	0.041
ASCVD, *n* (%)	2.54 (1.55–4.17)	<0.001
Cancer, *n* (%)	1.15 (0.69–1.90)	0.6
Chronic heart failure, *n* (%)	1.87 (0.92–3.80)	0.085
COPD, *n* (%)	1.66 (0.83–3.34)	0.2
Liver disease, *n* (%)	1.73 (0.78–3.85)	0.2

PIR, ratio of family income to poverty; BMI, body mass index; ASCVD, arteriosclerotic cardiovascular disease; COPD, chronic obstructive pulmonary disease.

### 3.3 Subgroup analysis

**Figure 5:**
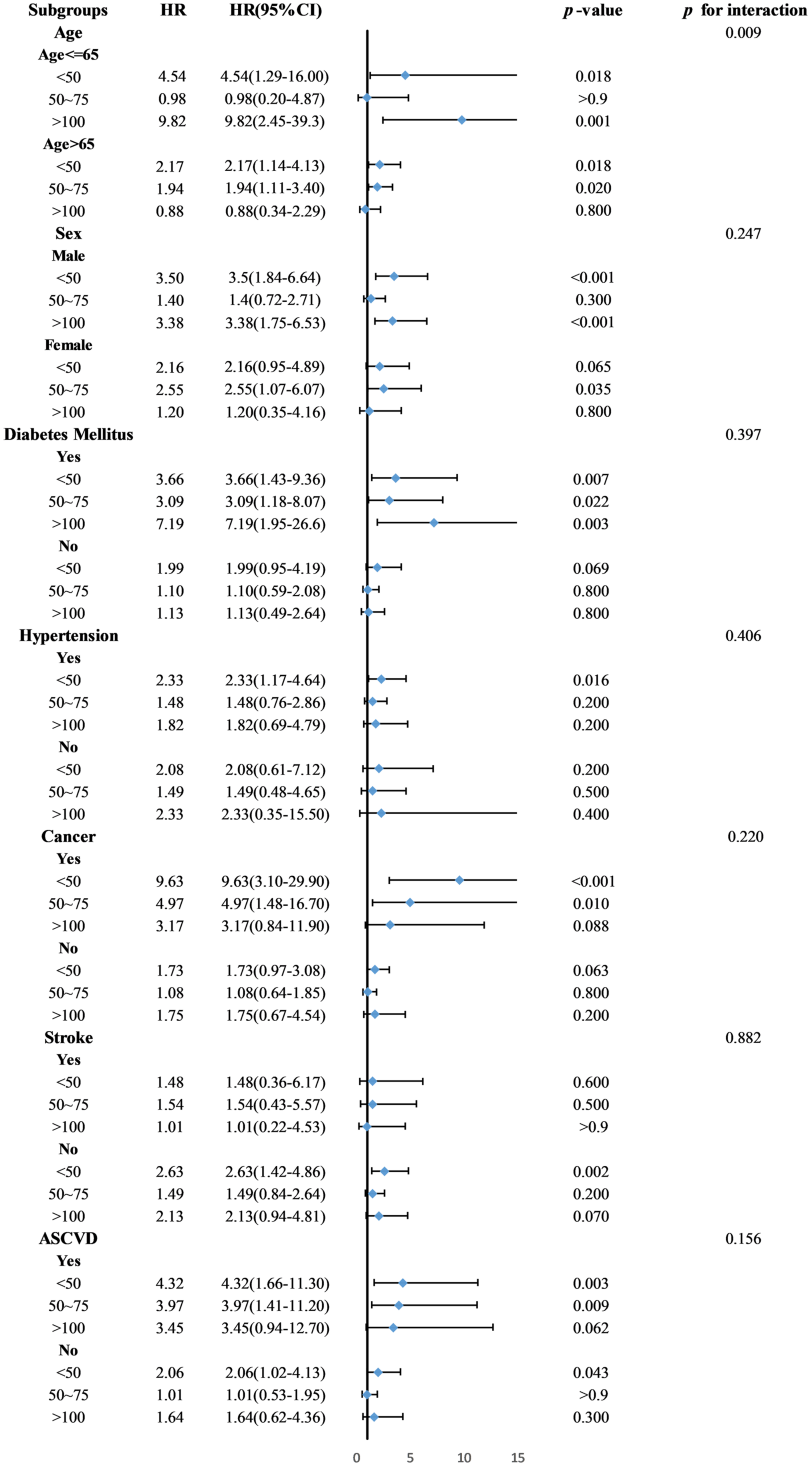
Subgroup analysis of the association between 25(OH)D and mortality.

Exploratory subgroup analyses indicated significant interactions between age and serum 25(OH)D levels (*p* = 0.009). In the subgroup aged ≤65 years, those in Groups 1 and 4 showed increased mortality risks with hazard ratios (HR) of 4.54 (95% CI = 1.29–16.0, *p* = 0.018) and 9.82 (95% CI = 2.45–39.3, *p* = 0.001), respectively. Among participants older than 65, increased risks were found in Groups 1 and 2, with HRs of 2.17 (95% CI = 1.14–4.13, *p* = 0.018) and 1.94 (95% CI = 1.11–3.40, *p* = 0.02). No significant interactions were observed for sex or comorbidities (Figure 5).

## 4 Discussion

This study established a U-shaped nonlinear relationship between serum 25(OH)D levels and mortality among PD patients, identifying the lowest mortality risk at approximately 78.68 nmol/L. PD patients with serum 25(OH)D concentrations within the optimal range of 75–100 nmol/L exhibited the highest survival rates. In contrast, those with levels below 50 nmol/L or above 100 nmol/L faced increases in mortality risk by factors of 3.52 and 2.92, respectively.

VD, a fat-soluble nutrient, undergoes transformation in the bloodstream to 1,25-dihydroxyvitamin D [1,25(OH)2D] through the action of 1α-hydroxylase, interacting with the vitamin D receptor (VDR) to influence gene transcription. Immunohistochemistry studies indicate a significant presence of 1α-hydroxylase and VDR in the dopaminergic neurons of the substantia nigra (22). VD plays a vital role in neuroprotection by promoting nerve growth factor production, regulating T-cell generation, reducing microglial activation, and decreasing the release of inflammatory factors, which helps in preventing the degeneration of dopaminergic neurons (23, 24).

Further supporting the importance of 25(OH)D, a Finnish cohort study reported a 65% reduced risk of developing PD in individuals with serum levels ≥50 nmol/L compared to those below 25 nmol/L. (17) Additionally, there is a correlation between 25(OH)D levels and PD motor symptoms. As PD progresses, indicated by higher Hoehn and Yahr stages, serum 25(OH)D levels tend to decrease, and there is a significant negative correlation with the Unified Parkinson’s Disease Rating Scale (UPDRS) scores (22, 25). Higher levels of 25(OH)D are also linked to better cognitive and mood outcomes in PD patients (26).

Serum 25(OH)D is derived from synthesis in the skin following sunlight exposure and from absorption through the gastrointestinal tract. Traditionally, it was believed that reduced outdoor activity led to insufficient 25(OH)D synthesis in PD patients. However, recent findings reveal a negative correlation between 25(OH)D2, primarily absorbed via the gastrointestinal tract and independent of sunlight, and the incidence of PD. This indicates that low serum 25(OH)D levels in PD patients may also stem from gastrointestinal dysfunction, affecting nutrient absorption (18). These insights suggest potential benefits from increasing oral VD supplementation in PD patients. Indeed, a 2023 cohort study noted that oral VD comprises 71% of dietary supplements used by PD patients, yet clinical guidelines for VD supplementation in this group remain undefined (27).

Previous guidelines on optimal serum 25(OH)D levels have varied. The Institute of Medicine suggests a level of 50 nmol/L suffices for 97.5% of the population, whereas the American Geriatrics Society recommends at least 75 nmol/L for older adults (16, 28).

Numerous studies have discussed appropriate serum 25(OH)D concentrations for different populations. A meta-analysis of 14 cohort studies has identified a U-shaped relationship between serum 25(OH)D concentrations and total mortality in the general population, pinpointing 75–87.5 nmol/L as the optimal range. Beyond this, the mortality reduction becomes insignificant (29). Studies in specific demographics, such as elderly men and postmenopausal women, have shown that mortality rates escalate with levels below 46 nmol/L or above 98 nmol/L, and with levels below a cutoff of 73.89 nmol/L, respectively (30, 31). Our research uniquely demonstrates that PD patients achieve the highest survival rates with serum 25(OH)D concentrations between 75–100 nmol/L, suggesting that levels outside this range could elevate the risk of mortality.

VD functions to mitigate excitotoxic damage by reducing cytoplasmic Ca^2+^ levels, decreasing nitric oxide synthase production, and lowering the formation of free radicals and reactive oxygen species (ROS). It also modulates immune responses by down-regulating cytokines, including interleukin-2 and tumor necrosis factor-α (32). Furthermore, increased serum 25(OH)D levels are linked to longer leukocyte telomere length (LTL), which is associated with longevity (33). VD enhances the apoptosis inhibitor Bcl-2, exerting anti-apoptotic effects by inhibiting death receptor-mediated apoptosis (34). Thus, it may decrease mortality through anti-inflammatory actions, modulation of LTL, and apoptotic signaling pathways. In subgroup analyses, age-related differences were observed concerning serum 25(OH)D levels and mortality risks in PD patients. Individuals aged ≤65 years with serum 25(OH)D concentrations below 50 nmol/L or above 100 nmol/L exhibited increased mortality risks. Conversely, in those over 65 years, levels below 75 nmol/L were associated with higher mortality, whereas levels above 100 nmol/L did not significantly impact mortality. These findings could guide VD supplementation strategies across various age groups.

This study has several limitations. This study, utilizing NHANES data, primarily reflects the health status of the American population, and results may not directly apply to other demographics. The public nature of the database limits access to detailed clinical assessments like UPDRS scores or Hoehn Yahr grading of PD patients, precluding more detailed analysis across different PD stages. Moreover, while the study controlled for known confounders, unidentified interfering factors cannot be completely ruled out. As an observational study, it does not establish causality between serum 25(OH)D levels and mortality in PD patients; thus, further higher-level research is needed to confirm these observations.

## 5 Conclusion

Findings from this study suggest that maintaining serum 25(OH)D levels within 75–100 nmol/L optimizes outcomes for PD patients. Those with levels below this range should consider increasing their VD intake to reach the recommended levels, while those above 100 nmol/L should adjust to maintain within this optimal range.

## Data availability statement

The original contributions presented in the study are included in the article/supplementary material, further inquiries can be directed to the corresponding author.

## Author contributions

YY: Writing – review & editing, Writing – original draft. HD: Writing – review & editing, Supervision, Conceptualization. ZZ: Writing – review & editing, Data curation, Formal analysis, Validation. YZ: Writing – original draft, Data curation, Formal analysis, Validation. MG: Writing – original draft, Investigation, Formal analysis. WL: Writing – original draft, Resources, Funding acquisition.
